# Pediatric Wilson’s disease: findings in different presentations. A cross-sectional study

**DOI:** 10.1590/1516-3180.2018.0210230718

**Published:** 2018-07-21

**Authors:** Şükrü Güngör, Mukadder Ayşe Selimoğlu, Fatma İlknur Varol, Serdal Güngör

**Affiliations:** I MD. Department of Pediatric Gastroenterology, Hepatology and Nutrition, İnönü Üniversitesi Tıp Fakültesi, Malatya, Turkey.; II MD. Professor, Department of Pediatric Gastroenterology, Hepatology and Nutrition, İnönü Üniversitesi Tıp Fakültesi, Malatya, Turkey.; III MD. Department of Pediatric Gastroenterology, Hepatology and Nutrition, İnönü Üniversitesi Tıp Fakültesi, Malatya, Turkey.; IV MD. Professor, Department of Pediatric Neurology, İnönü Üniversitesi Tıp Fakültesi, Malatya, Turkey.

**Keywords:** Hepatolenticular degeneration, Child, Zinc, Iron

## Abstract

**BACKGROUND:**

Wilson’s disease (WD) may present with different manifestations: from an asymptomatic state to liver cirrhosis. Here, we aimed to evaluate clinical presentations and laboratory findings and prognoses among WD cases.

**DESIGN AND SETTING:**

Cross-sectional study based on patients’ records from the university hospital, İnönü University, Malatya, Turkey.

**METHODS:**

The medical records of 64 children with WD were evaluated focusing on the clinical, laboratory and liver biopsy findings in different clinical presentations.

**RESULTS:**

The mean age at diagnosis was 8.6 ± 3.26 years (range 3.5-17) and mean length of follow-up was 2.49 years (range 0-9). There were 18 cases (28.1%), 12 (18.8%), 9 (14.1%) and 6 (9.4%) of chronic liver disease, fulminant liver failure, neurological WD and acute hepatitis, respectively. Nineteen (29.7%) were asymptomatic. The most common sign and laboratory finding were jaundice (45.3%) and hypertransaminasemia (85.9%), respectively. The lowest serum zinc level was found in the fulminant liver failure group (P = 0.035). Hepatosteatosis was detected in 35% of the 20 patients who underwent liver biopsy. Among those with hepatosteatosis, 57.1% were asymptomatic. While 35% had copper staining, 25% presented iron accumulation in liver biopsies. Nine cases underwent liver transplantation and seven of these presented fulminant liver failure (77.8%).

**CONCLUSION:**

The presentation, symptoms and signs of our cases were similar to those in previously reported series, except for the high proportion of fulminant WD cases. Further studies are needed to clarify the relationship between zinc levels and development of a fulminant course and between iron status and WD.

## INTRODUCTION

Wilson’s disease (WD) is a rare autosomal recessive disorder of copper metabolism that occurs due to mutations in the copper-transporting ATP7B gene. Impaired excretion of copper through bile and decreased incorporation into ceruloplasmin cause excessive copper accumulation in different organs, including the liver, brain and cornea.^1^^,^^2^^,^^3^^,^^4^^,^^5^ WD typically begins with a presymptomatic period, during which copper accumulation in the liver causes subclinical hepatitis, and progresses to liver cirrhosis and development of neuropsychiatric symptoms. There is no single diagnostic test that can rule out or confirm WD with certainty. Because of the lack of such a test, the diagnosis needs to be based on a combination of clinical features and laboratory, neuroradiological and pathological findings and also on the results from genetic analysis.^5^^,^^6^^,^^7^^,^^8^^,^^9^^,^^10^

Pharmacological treatment of WD has the aim of preventing further accumulation of copper, through either reducing its absorption or promoting its excretion in urine or bile, or both.^5^^,^^6^ The indications for liver transplantation to cure WD include progressive liver failure, progressive neurological symptoms, complications from portal hypertension (despite medical and dietary therapy) and acute liver failure.^5^^,^^6^

There are not many detailed studies investigating pediatric WD series in the literature. ^5^^,^^6^^,^^7^^,^^8^^,^^9^^,^^10^ In the present study, we aimed to evaluate clinical presentations and laboratory findings among 64 children who were diagnosed with WD in our clinic over a nine-year period, and to determine their response to treatment and their prognosis from the follow-up.

## METHOD

### Study design, setting and ethical issues

This was a cross-sectional study based on retrospective evaluation of data from medical records, undertaken in the Pediatric Gastroenterology and Nutrition Department, İnönü Üniversitesi Tıp Fakültesi, Malatya, Turkey. Prior to the start of the work, local institutional ethics committee approval was obtained, as follows: ethics committee date: 2017; and ethics committee no: 22-2.

### Participants

The medical records of 64 consecutive cases of pediatric WD that were diagnosed in the Pediatric Gastroenterology and Nutrition Department of our institution between January 2006 and December 2015 were evaluated.

The patients included were those who presented with elevated transaminases, acute or chronic liver failure or neuropsychiatric symptoms, or those who were asymptomatic siblings of index cases. The diagnosis of WD was based on the scoring system developed at the Eighth International Meeting on Wilson’s disease, in Leipzig, 2001; a score > 4 was considered reasonable for making a diagnosis of WD.^6^^,^^11^ No data on genetic mutational analysis could be provided. Other causes of liver disease such as autoimmune liver disease, chronic viral hepatitis, α1-anti-trypsin deficiency or other metabolic conditions were ruled out in all cases. Detailed follow-up data were collected in relation to each patient.

The clinical presentations were defined as follows:^5^^,^^6^^,^^12^^,^^13^

Asymptomatic WD: Symptom-free patients with or without hypertransaminasemia and/or hepatomegaly who had been diagnosed during family screening or routine check-up.

Acute WD: Acute-onset clinically and biochemically diagnosed liver disease in patients without any previously recognized liver disease.

Fulminant WD: Acute liver failure was defined in accordance with the criteria of the Pediatric Acute Liver Failure Study Group:

1. children with no evidence of chronic liver disease;
2. biochemical evidence of acute liver injury; or
3. hepatic-based coagulopathy.

This last criterion was defined as prothrombin time (PT) ≥ 15 s or international normalized ratio (INR) ≥ 1.5 that was not corrected by means of vitamin K in the presence of hepatic encephalopathy; or as PT ≥ 20 s or INR ≥ 2.0, regardless of the presence or absence of clinical encephalopathy.

Chronic liver disease: Patients with previously known/unknown WD who presented with clinical and laboratory findings of chronic liver failure.

Neurological WD: Patients presenting with neurological symptoms.

Histopathological examinations were performed on explant liver tissues from patients who underwent liver transplantation, and on percutaneous samples that had been provided from some patients.

### Statistical analyses

We did not analyze the study power because all the patients in our group were evaluated retrospectively.

The data were analyzed statistically using the Statistical Package for the Social Sciences for Windows, version 16.0 (SPSS Inc, Chicago, USA). The continuous variables were reported as the mean ± standard deviation, whereas the categorical variables were defined as percentages. The data were tested for normal distribution using the Kolmogorov-Smirnov test. To compare the continuous variables, Student’s t test, the one-way analysis of variance test or the Kruskal-Wallis test was used, as appropriate. When significant differences were observed between the groups based on the post-hoc analyses, either the Tukey or the Scheffe test was used to determine the differences between the groups. The chi-square test was used to compare the categorical variables. Statistical significance was defined as P < 0.05.

## RESULTS

Out of the 64 patients evaluated, 39 (60.9%) were boys and 25 (39.1%) were girls. Their mean age at the time of the diagnosis was 8.6 ± 3.26 years (range 3.5-17 years) and the mean length of follow-up was 2.49 years (range 0-9 years). While the most common manifestation of the disease was jaundice (45.3%), the most common clinical findings were jaundice and splenomegaly (45.3%) followed by hepatomegaly (43.8%) and Kayser-Fleischer ring (KF) (40.6%) (Table 1). Consanguinity and a familial history of WD were detected in 46 patients (71.9%) and 32 (50%), respectively. Among all the patients, 16 received the diagnosis through family screening (25%). Some of the clinical and laboratory findings are shown in Table 1.

**Table 1:** Some special clinical and laboratory findings regarding the presentation of Wilson’s disease

Features	Asymptomatic 19	Acute 6	Chronic 18	Fulminant 12	Neurological 9	Total	P
	N (%)	N (%)	N (%)	N (%)	N (%)	N (%)	
Kayser-Fleischer ring	4 (21)	2 (33.3)	9 (50)	2 (16.6)	9 (100)	26 (40.6)	0.00
Hemolytic anemia	0 (0)	0 (0)	2 (11.1)	7 (58.4)	0 (0)	9 (14)	0.00
Hypouricemia	5 (26.3)	1 (16.6)	14 (77.8)	12 (100)	5 (55.6)	37 (57.8)	0.00
Low albumin	3 (15.8)	2 (33.3)	11 (61.1)	11 (91.7)	1 (11.1)	28 (43.7)	0.00
High INR	3 (15.8)	0 (0)	14 (77.8)	12 (100)	3 (33.3)	32 (50)	0.00
High ammonium	0 (0)	0 (0)	5 (27.8)	12 (100)	0 (0)	17 (26.5)	0.00
Low HDL	5 (26.3)	1 (16.6)	12 (66.7)	10 (83.4)	4 (44.5)	32 (50)	0.03
Normal ceruloplasmin	2 (10.5)	1 (16.6)	3 (16.6)	0 (0)	1 (11.1)	7 (11)	0.41
Hepatosteatosis (n = 57) (USG)	2/15 (13.3)	4/6 (66.6)	0/17 (0)	1/11 (9)	0/8 (0)	7/57 (12.3)	0.00
Brain involvement (n = 41) (MRI)	1/6 (16.6)	0 (0)	11/14 (79)	5/8 (62.5)	9/9 (100)	26/41 (63.4)	0.00
Steatosis in biopsy (n = 20)	4/5 (80)	2/3 (66.6)	0/4 (0)	1/7 (14.3)	0/1 (0)	7/20 (35)	0.02
Iron accumulation in biopsy (n = 20)	0/5 (0)	0/3 (0)	2/3 (66.6)	3/7 (43)	0/1 (0)	5/20 (25)	0.11
Family history	14 (87.5)	3 (50)	6 (33.3)	4 (33.3)	5 (55.5)	32 (50)	0.103
Detected through screening	13 (68.4)	0	1 (5.5)	0	2 (16.7)	16 (25)	0.000
Hepatomegaly	3 (15.7)	4 (66.7)	17 (94.5)	10 (83.4)	2 (22.3)	36 (56.2)	0.000
Splenomegaly	1 (5.2)	1 (16.7)	15 (83.4)	9 (75)	3 (33.3)	29 (45.3)	0.000
Ascites	0	1 (16.7)	9 (50)	7 (58.3)	2 (22.3)	19 (29.6)	0.002
Jaundice	0	3 (50)	14 (77.8)	12 (100)	0	29 (45.3)	0.000
Encephalopathy	0	0	0	12 (100)	0	12 (18.7)	0.000

INR = international normalized ratio; HDL = high-density lipoprotein; USG = ultrasonography; MRI = magnetic resonance imaging.

Evaluation on the patients’ clinical presentations showed that 19 (29.7%) were asymptomatic; and that 18 (28.1%), 12 (18.8%), 9 (14.1%) and 6 (9.4%) had chronic liver disease, fulminant liver failure, neurological WD and acute hepatitis, respectively. Among all the children, 19 (26%) presented both positive brain magnetic resonance imaging (MRI) findings and chronic liver disease findings. MRI examination revealed brain involvement in 26 out of 41 patients (63.4%); nine of them (34.6%) had symptomatic neurological WD and, among the remaining 17 patients, 11 (64.7%) had chronic liver disease, five (29.4%) had fulminant liver failure and one (5.8%) was asymptomatic at the time of the diagnosis. An association between brain MRI findings (intensity increase in basal ganglia) and chronic liver disease presentation was significant (P < 0.0001), while between asymptomatic WD and fulminant WD it was not.

Twenty-six patients (40.6%) presented KF rings. This was most common in cases of neurological WD (100%) and least common in cases with fulminant liver failure (16.6%) (Table 2). KF was detected in 18 of the 21 patients (85.7%) who had MRI-detected brain involvement (Table 1).

**Table 2:** Laboratory findings at the time of diagnosis according to the type of presentation

Laboratory findings	Asymptomatic	Acute	Chronic	Fulminant	Neurological	Total	P
	Mean (Range)	Mean (range)	Mean (range)	Mean (range)	Mean (range)	Mean (range)	
WBC (10^3^/ml)	7.4 (5-11.5)	10.2 (7.5-13.7)	6.5 (2.3-14.8) β	12 (4.4-34) α	5.3 (2.9-8.1) β	8 (2.3-34)	0.007
INR	1 (0.9-1.7) β	1 (1-1.3) β	2.2 (1-4.5) β	4.5 (2.5-8) α	1.48 (1-2.9) β	2.1 (0.9-8)	0.000
AST (U/l)	132.4 (20-302)	164.6 (82-400)	175.2 (23-609)	362.4 (66-1307) α	50.3 (29-95) β	182.7 (20-1307)	0.014
ALT (U/l)	187.2 (27-450)	184.5 (103-272)	120.2 (16-384)	259.2 (23-1345)	33.5 (13-79)	161.2 (13-1345)	0.095
AST/ALT	0.79 (0.5-1.5)	0.98 (0.34-2.03)	1.4 (0.85-2.45)	2.73 (1.09-9.1)	1.75 (1.16-2.61)	1.49 (0.34-9.1)	0.212
ALP (U/l)	559.5 (84-4042)	330.3 (202-514)	290.7 (57-537)	216 (19-511)	281 (129-613)	353.5 (19-4042)	0.394
T. bil (mg/dl)	0.5 (0.2-1) β	2 (0.2-5) β	6.8 (0.2-26.9) β	22.3 (1.9-43.8) α	0.7 (0.5-1) β	6.8 (0.2-43.5)	0.000
D. bil (mg/dl)	0.2 (0.1-0.4) β	0.66 (0.1-2) β	4.7 (0.1-23.6) β	14.8 (1-29) α	0.3 (0.1-0.7) β	4.4 (0.01-29)	0.000
Albumin(mg/dl)	3.9 (2.8-4.9) β	3.5 (2.4-4)	3 (1.7-5.8)	2.5 (1.8-3.7) α	3.8 (2.4-4.5) β	3.3 (1.7-5.8)	0.000
Uric acid (mg/dl)	2.5 (1-4) β	2.6 (1-3.6) β	1.5 (0.3-4)	0.9 (0.2-1.8) α	1.4 (0.5-2.5)	1.7 (0.2-4)	0.000
Zinc (mg/dl)	79.9 (46-128) β	80 (80) β	56.6 (28-70)	52 (46-56) α	60.2 (36-75)	66.5 (28-128)	0.035
Ferritin (ng/ml)	56.7 (10.2-125) β	39.5 (36-43)	163.2 (15.7-604)	600.4 (94-1500) α	53.6 (18.4-81) β	193.1 (10.2-1500)	0.038
Ceruloplasmin (g/l)	0.1 (0-0.3)	0.12 (0.2-2)	0.12 (0.1-0.4)	0.11 (0.01-0.19)	0.15 (0.05-0.5)	0.11 (0.00-0.5)	0.814
24-hour urinary Cu (µg/24 h)	329.6 (8-1287) β	368.3 (52-1319)	879.8 (25-4480)	1546.7(18-5280) α	521.7 (265-1066)	758.1 (8-5280)	0.047
24-hour urinary Cu after d-penicillamine (µg/24 h)	1153 (161-1984) β	1322 (170-4000)	833.5 (308-1120)	1495 (367-4096) α	1060 (1060)	1191 (161-4096)	0.93
Liver Cu level (µg/g)	855.6 (250-1244)	423 (275-510)	676.8 (277-1310)	-	292(292)	672 (250-1310)	0.31
Mortality score	2.76 (0-5) β	6.1 (3-14) β	9.3 (1-18) β	14.25 (11-17) α	2.5 (1-7) β	7.2 (0-18)	0.000

P < 0.05 values signify comparison of α to β in one-way analysis of variance. WBC = white blood cell; INR = international normalized ratio; AST = aspartate aminotransferase; ALT = alanine aminotransferase; ALP = alkaline phosphatase; T. bil = total bilirubin; D. bil = direct bilirubin; Cu = copper.

In 16 out of the 33 cases (48.5%) that underwent upper gastrointestinal endoscopy, esophageal varices were found.

The most common laboratory finding consisted of elevated transaminase levels (85.9%). Coombs-negative hemolytic anemia was detected in nine cases (14.8%): seven (77.8%) presented with fulminant liver failure whereas two (22.2%) had findings relating to chronic liver disease. There was a significant association between occurrences of fulminant liver failure and Coombs-negative hemolytic anemia (P < 0.0001).

Assessment of the association between laboratory findings and the clinical presentation showed that total protein, alanine aminotransferase (ALT), alkaline phosphatase (ALP) and ceruloplasmin levels were not significantly different according to presentation. However, the levels of albumin, aspartate aminotransferase (AST), total bilirubin, direct bilirubin, uric acid, white blood cells (WBCs), INR, 24-hour urinary copper and zinc differed significantly among the groups (Table 2).

One year after the treatment, the AST, ALP, zinc and 24-hour urinary copper levels were not significantly different between the groups, but the albumin, ALT, total bilirubin, direct bilirubin, uric acid and INR levels were still different.

All of the fulminant cases had low levels of ceruloplasmin, whereas seven cases (11.3%) with other presentations had normal levels. Out of all the cases, four (6.4%) had normal levels of urinary copper whereas all of the neurological WD cases had high urinary copper levels.

Blood ferritin levels were significantly higher in fulminant WD cases than in asymptomatic and neurological WD cases (P = 0.038).

Liver samples from 20 patients were stained to ascertain iron levels; only five of these patients (25%) had iron deposits. Two of these patients (40%) presented with chronic liver disease and three (60%) with fulminant WD (Table 1). Hepatosteatosis was found in seven cases (35%): four of these cases (57.1%) were asymptomatic, two (28.5%) had acute hepatitis and one (14.2%) had fulminant liver failure at the time of the diagnosis (Table 1).

Nine cases underwent liver transplantation. Among these, seven had fulminant liver failure (77.8%), one had neurological WD (11.1%) and one had chronic hepatitis (11.1%) at the time of the diagnosis.

D-penicillamine and trientine were used for 23 and 14 patients, respectively, during the first year of treatment. For 18 children, their use of d-penicillamine was switched to trientine because of side effects or problems in obtaining the agent. Comparison of alterations to laboratory parameters between the d-penicillamine and trientine groups showed that the increase in serum albumin levels was significantly greater with trientine treatment (P = 0.013).

According to the mortality score of Dhawan et al.,^12^ 21 patients had a mortality score of > 11. Twelve of those patients had fulminant hepatitis (57.2%), eight had chronic hepatitis (38%) and one had acute hepatitis (4.8%) at presentation. Only seven of those patients (30%) underwent liver transplantation. Two patients with a score of < 11 underwent transplantation: one of them had neurological WD and the other had chronic hepatitis at presentation. The sensitivity, specificity, positive and negative predictive values and accuracy of this index were detected as 77.78%, 74.5%, 33.34%, 95.3% and 75%, respectively.

## DISCUSSION

Studies on childhood WD present limitations because the number of patients available to make up a relevant study group is frequently low, which precludes large cohort studies or randomized controlled trials. Machado et al.^14^ evaluated 119 Brazilian patients, mostly adults, with a mean age of 19.3 at the time of diagnosis and reported that the major initial clinical manifestation was neurological. Our study is the most detailed single-centered study investigating the clinical and laboratory findings at admission and over the follow-up, among quite a large number of children with WD. The mean age (8.6 ± 3.26 years) and male dominance (60.9%) among our subjects were compatible with what has been described in the literature.^9^ It has been reported that 10-33% of the cases detected through family screening were asymptomatic;^7^^,^^9^^,^^10^ half of our cases had a family history and one-quarter of the cases were diagnosed through family screening.

There is no single diagnostic test that can rule out or confirm WD with certainty. A diagnosis of WD is based on a combination of clinical features, laboratory findings and genetic testing, if available.^9^^,^^15^^,^^16^ In our study, the diagnosis of WD was based on the scoring system developed at the Eighth International Meeting on Wilson’s disease, in Leipzig, 2001.^6^^,^^11^ Genetic mutational analysis data could not be provided.

We found that jaundice was the most common symptom on physical examination (45.3%), along with organomegaly (Table 1), which was consistent with reports in the literature.^9^ Rukunuzzaman et al.^7^ reported that 69% of 100 WD cases presented as hepatitis, 14% had both hepatic and neurological symptoms, 1% had psychiatric symptoms and 10% were asymptomatic. Similarly, most of our patients presented with hepatic symptoms (56.2%), but nearly one third of our patients were asymptomatic (29.7%) and none presented with psychiatric symptoms. In the abovementioned study,^7^ among the hepatic presentations, 76% consisted of chronic hepatitis and 4% consisted of fulminant liver failure at presentation.^7^ In our series, fulminant presentations were detected in 18.8% of all the cases: this was quite a high number and it might be attributable to the fact that our hospital is the largest liver transplantation center in the country.

We observed KF rings in 40.6% of all patients. Their presence has previously been reported in 38%-77%, most commonly in those with neurological WD (85-90%), followed by those with chronic hepatitis (52%-84%).^5^^,^^7^^,^^9^^,^^10^ It was interesting to find a higher percentage of presence of KF rings in asymptomatic children than in the fulminant liver failure group (21% versus 16.7%) in our study. KF rings were present in 100% of the children with neurological WD, and this was consistent with the classical knowledge.

Normal ceruloplasmin levels, which were observed in 10.9% of our cases, have previously been reported in 10%-36% of the cases.^5^^,^^7^^,^^9^^,^^10^^,^^17^ While the ratio was similar among different presentations, it was remarkable that none of the fulminant liver failure cases had normal ceruloplasmin levels.

Magalhães et al.^18^ reported that three of their 11 patients with WD (27%) had MRI findings without any clinical sign. We found that this ratio was 42.1% (8/19 patients) in our study. It is still not clear when the brain involvement becomes symptomatic and there is no consensus regarding use of MRI screening for neurological involvement in asymptomatic cases.

Twenty-four-hour urinary copper levels were reported to be higher than 200 µg in 99% of the patients after d-penicillamine challenge in a previous study.^7^ El-Karaksy et al.^19^ found that 53% of the cases had urinary copper > 100 µg and that after d-penicillamine challenge, 15% of them had urinary copper < 1000 µg/day. In our study, 6.4% of the cases had levels lower than 40 µg/day. Among the patients who underwent d-penicillamine challenge, 9% had urinary copper < 200 µg/day despite high liver copper content. Twenty-four-hour urinary copper levels were significantly higher in patients presenting with fulminant liver failure than in asymptomatic patients (P = 0.036). These findings might suggest that urinary copper levels are related to clinical presentation and that the d-penicillamine challenge test might not be 100% reliable for making diagnoses.

Rukunuzzaman et al.^7^ reported that the ALT levels were elevated in 92% of their patients and that the mean AST levels were higher than the ALT levels in their study.^7^ It was also shown that the AST/ALT ratio was > 2.2 in the patients who presented with fulminant hepatitis.^5^^,^^7^ Our data supported the findings of the abovementioned studies, with higher AST than ALT and an AST/ALT ratio of 2.73 in the patients with fulminant hepatitis (Table 2). Iorio et al.^8^ reported that the ALT level might be normal in 9.5%; it was normal in 14% in our series.

A wide range of serum albumin and bilirubin levels has been reported in the literature.^7^^,^^19^ In our fulminant cases, the mean albumin and cholesterol levels were lower, as expected, because of the impaired capacity of the liver for synthesis. Interestingly, the mean serum zinc level and 24-hour urinary copper level were significantly higher than in other presentations, which suggested that high levels of urinary copper and, especially, low levels of zinc might affect progression to fulminant liver failure. In a recent case report, a 2.5-year-old child with clinical signs of both Zn deficiency and WD was presented with the emphasis of the close relationship between Cu and Zn.^20^ In a cohort of 18 children with WD, significant decrease in serum plasma Zn has been previously described.^20^ Unfortunately, in that paper, it was not clear whether any of the patients had fulminant presentation or not.^21^ It was also reported that in a subgroup of WD population with a mild liver disease, Zn serum level was normal or high at diagnosis.^22^ These observations suggest that Zn serum levels could be related with clinical phenotype of patients with WD and its severity.^22^

The early histological findings in WD cases comprise micro or macrovesicular steatosis, glycogen accumulation in hepatocyte nuclei and focal hepatocellular necrosis.^5^ Monolaki et al.^9^ reported the presence of hepatosteatosis in 50% of their symptomatic and 87% of their asymptomatic patients. Its presence was reported as 54% and 24% in other two studies.^8^^,^^10^ Hepatosteatosis was present in one-third of the 20 patients from whom hepatic specimens were available. 57% of these patients were asymptomatic. This finding emphasizes the importance of considering WD as a diagnosis in situations of incidentally found hepatosteatosis.

Another interesting finding was the iron accumulation in one-quarter of the patients from whom a liver biopsy had been obtained. There are only a few studies regarding iron accumulation in WD cases.^23^^,^^24^ Shiono et al.^23^ showed that histopathological iron accumulation occurred in two out of four cases before treatment and in all of them after treatment. They observed hypertransaminasemia and concurrent elevation of ferritin levels, which started to decrease after phlebotomy.^23^ Another study revealed histological iron accumulation in seven out of ten patients, all of whom had hyperferritinemia. The authors of that study emphasized the possibility that cases might worsen after treatment.^24^ In our study, three fulminant and two chronic hepatitis cases had histological iron accumulation. Three of them underwent liver transplantation and one of them had hyperferritinemia.

Dhawan et al.^17^ prospectively applied a new Wilson mortality score, which showed that patients scoring ≥ 11 needed liver transplantations. Accordingly, 21 of our cases scored ≥ 11. However, only seven of these patients needed transplantation and there were two patients who underwent transplantation despite a score < 11: one with neurological WD and the other with chronic hepatitis. Although this scoring system was developed using data on children with WD who either survived or died, we used it for those who survived, those who died or those who underwent liver transplantation, and then grouped the latter two categories together. This was because the indication for liver transplantation in our series was presentation of decompensation findings that had been unresponsive to treatment. The lower sensitivity and specificity that we detected might have been due to the effectiveness of plasmapheresis in some cases with fulminant presentation in our series.

## CONCLUSIONS

The presentation, symptoms and signs of our cases were similar to those in previously reported series, except for the high proportion of fulminant WD cases, which probably occurred because our institution is a liver transplantation center. Although the data were insufficient to conclude that decreased zinc levels have a role in the development of a fulminant course, we emphasize the need for further studies on this topic. We also emphasize the importance of incidentally detected hepatosteatosis in making an early diagnosis of WD and the probable relationship between iron status and WD.
